# An Apology

**Published:** 1876-06

**Authors:** 


### Editorial.

---

An Apology.—Circumstances over which we had no control have delayed the issue of the present number of the Journal. We promise our readers that this shall not again occur.

